# Detection properties of indium-111 and IRDye800CW for intraoperative molecular imaging use across tissue phantom models

**DOI:** 10.1117/1.JBO.30.S1.S13705

**Published:** 2024-09-20

**Authors:** ReidAnn E. Sever, Lauren T. Rosenblum, Kayla C. Stanley, Angel G. Cortez, Dominic M. Menendez, Bhuvitha Chagantipati, Jessie R. Nedrow, W. Barry Edwards, Marcus M. Malek, Gary Kohanbash

**Affiliations:** a University of Pittsburgh, Department of Neurological Surgery, Pittsburgh, Pennsylvania, United States; b University of Pittsburgh, Department of Surgery, Pittsburgh, Pennsylvania, United States; c University of Pittsburgh School of Medicine, Pittsburgh, Pennsylvania, United States; d University of Pittsburgh Medical Center, In Vivo Imaging Facility Core, Hillman Cancer Center, Pittsburgh, Pennsylvania, United States; e University of Missouri, Department of Biochemistry, Columbia, Missouri, United States; f University of Pittsburgh School of Medicine, Division of Pediatric General and Thoracic Surgery, Department of Surgery, Pittsburgh, Pennsylvania, United States; g University of Pittsburgh, Department of Immunology, Pittsburgh, Pennsylvania, United States

**Keywords:** intraoperative molecular imaging, tissue phantom, fluorescence, radioactivity, tumor detection, three-dimensional printing

## Abstract

**Significance:**

Intraoperative molecular imaging (IMI) enables the detection and visualization of cancer tissue using targeted radioactive or fluorescent tracers. While IMI research has rapidly expanded, including the recent Food and Drug Administration approval of a targeted fluorophore, the limits of detection have not been well-defined.

**Aim:**

The ability of widely available handheld intraoperative tools (Neoprobe and SPY-PHI) to measure gamma decay and fluorescence intensity from IMI tracers was assessed while varying characteristics of both the signal source and the intervening tissue or gelatin phantoms.

**Approach:**

Gamma decay signal and fluorescence from tracer-bearing tumors (TBTs) and modifiable tumor-like inclusions (TLIs) were measured through increasing thicknesses of porcine tissue and gelatin in custom 3D-printed molds. TBTs buried beneath porcine tissue were used to simulate IMI-guided tumor resection.

**Results:**

Gamma decay from TBTs and TLIs was detected through significantly thicker tissue and gelatin than fluorescence, with at least 5% of the maximum signal observed through up to 5 and 0.5 cm, respectively, depending on the overlying tissue type or gelatin.

**Conclusions:**

We developed novel systems that can be fine-tuned to simulate variable tumor characteristics and tissue environments. These were used to evaluate the detection of fluorescent and gamma signals from IMI tracers and simulate IMI surgery.

## Highlights

1. Detection of indium-111 and IRDye800-CW in tracer-bearing tumors (TBTs) and tumor-like inclusions (TLI) through porcine and gelatin phantoms was measured using widely available handheld instruments for intraoperative molecular imaging (IMI).
2. Customized 3D-printed molds were used to precisely measure the detection properties of tracers, with the aim of improving complete surgical resection.
3. These tracer/phantom models can be used to characterize intraoperative tracers as well as to train surgeons in the practice of IMI.

## 1 Introduction

Surgical resection is a critical component of solid cancer treatment and has significant implications on local recurrence and survival rates.^1^^–^^3^ Despite this, the rate of inadequate surgery and complications remains high.^1^^–^^7^ For decades, surgeons have depended upon preoperative imaging, vision, and palpation to detect and remove tumors, though these methods may lack the necessary precision for complete and safe resection.^8^^,^^9^ Although preoperative imaging continues to evolve and improve, lesions can be missed and intraoperative findings may not correspond as expected due to overlying tissues and movement of organs, leading to difficulty locating tumors and/or unclear margins.^5^^,^^10^^,^^11^ Difficulty in distinguishing tumor margins also contributes to resection of normal tissue or incomplete resection.^10^ The rate of positive margins varies widely depending on tumor type, typically between 15% and 40% for various abdominal cancers, and are correlated with worse local recurrence, shorter event-free survival, and shorter overall survival.^2^^,^^4^^,^^12^^–^^14^ Incomplete resection of neuroblastoma, for example, occurs in 30% of patients and leads to increased local recurrence with decreased event-free and overall survival.^1^^,^^2^

Current intraoperative imaging adjuncts include ultrasound, radio-guided surgery (RGS), and fluorescence-guided surgery (FGS). Sentinel lymph node biopsy using methylene blue or RGS with Tc-99m, for example, has provided clear benefits for patients with breast cancer and melanoma.^15^ Those methods, however, depend on anatomical differences and flow of tracer, but are not specific to the tumor.^16^^–^^19^ The next generation of surgeons and patients will benefit from sensitive and specific tumor-targeted intraoperative guidance. IMI is a rapidly expanding field that utilizes tumor-specific FGS and RGS to precisely detect cancer. Numerous targeted RGS tracers are in development, for cancers including pancreas, prostate, colorectal, and ovarian.^20^^–^^23^ RGS enables excellent depth of detection but poor visualization of margins. By contrast, FGS has a relatively poor depth of detection but excellent visualization of margins. Many FGS tracers are also in preclinical and clinical trials, and the first targeted fluorescent tracer, OTL38, was recently U.S. Food and Drug Administration (FDA)-approved for IMI of metastatic ovarian cancer and lung cancer.^24^^,^^25^ FGS yields fantastic visualization of tumors with a sharp delineation from normal tissue, but detection of fluorescence is limited by overlying tissue. Dual-labeled agents incorporate the benefits of RGS and FGS, leading to excellent depth of detection as well as tumor visualization. Accordingly, the preclinical development of dual-labeled tracers for cancer has garnered interest with one tracer, In111-DOTA-girentuximab-IRDye800CW, in phase I clinical trials for resection of clear cell renal cell carcinoma.^23^

Despite the extensive development of fluorescent and radioactive tracers, limited data exists regarding their limits of detection. Porcine tissue has occasionally been used as a proxy for human tissue to study imaging and detection methods, though most studies of detection parameters use synthetic phantoms.^26^^–^^29^ While phantom studies have been used extensively in PET and SPECT research, little work exists for detection parameters in RGS.^30^^,^^31^ For FGS, gelatin, agarose, or polymer phantoms can be used to simulate the thickness and optical properties of tissue, through the addition of specific compounds.^32^ Scatter of tissues can be simulated with the inclusion of lipids, polymer microspheres, ${\mathrm{TiO}}_{2}$ or ${\mathrm{Al}}_{2}O_{3}$ powders, or quartz glass microspheres, while absorption can be simulated with blood/hemoglobin, ink, or dyes, each with advantages for various studies and modifiable to consider different tissue types.^32^^,^^33^ Molds can be used to generate specific shapes such as those of organs to better simulate intraoperative parameters, but their creation is burdensome and not easily modifiable.^33^^,^^34^ TLIs, typically made from solidified gelatin or agarose solutions, have also been generated and embedded in phantom tissue to assess the impact of tumor characteristics on IMI properties, though precise generation and placement are limited.^35^^,^^36^

We recently created a dual-labeled anti-GD2 IMI tracer for neuroblastoma (DTPA[In-111]-antiGD2-IR800), as rates of complications and inadequate resection are 30% or greater.^1^^,^^2^ Our tracer was developed for detection by commonly used surgical tools, the Neoprobe^®^ handheld gamma detector (for indium-111 detection), and the SPY-PHI handheld fluorescent camera (for IRDye800CW imaging).

The aim of this work was to generate and use precisely defined phantom models to assess the properties and parameters of IMI. To evaluate the detection of indium-111 and IRDye800CW with the Neoprobe^®^ and SPY-PHI tools, we assessed signals from TBTs and tumor-like-inclusions (TLIs) through porcine tissue and gelatin phantoms, using precisely generated 3D-printed molds. Although we specifically studied the detection of indium-111 with the Neoprobe^®^ and IRDye800CW with the SPY-PHI camera, these methods could easily be modified to test other tracers and IMI instruments.

## 2 Materials and Methods

### 2.1 Tumor Xenografts

Animal studies were performed in accordance with the Institutional Animal Care and Use Committee (IACUC) of the University of Pittsburgh (protocol number: 22101912). Four- to 6-week-old athymic nude mice ($Fox1^{nu}$, Jackson Labs, strain #002019) were maintained in a temperature-controlled animal facility at the UPMC Hillman Cancer Center. Tumors were grafted as previously described.^37^^–^^39^ One million SK-N-BE(2) cells suspended in 20  μL of PBS and 20  μL of Matrigel (Corning™ #354234) were implanted into the surgically exposed left adrenal gland of anesthetized mice. After 5 weeks of tumor growth, mice were given tail vein injections of DTPA[In-111]-antiGD2-IR800 (except in the autofluorescence experiment). Four days later, mice were euthanized, and tumors and organs were then harvested, including the heart, lungs, blood, spleen, liver, right kidney, left kidney, intestines, muscle, right adrenal gland, brain, and bone.

### 2.2 Tracer Synthesis and Evaluation

To generate DTPA-antiGD2-IR800, antiGD2 antibody (Clone 14G2a, Bio X Cell # BE0318) was incubated with ∼25-fold molar excess of p-SCN-Bn-CHX-A”-DTPA (Macrocyclics) for 1 h and purified. DTPA-antiGD2 was then incubated with ∼four-fold molar excess of IRDye800CW-NHS ester (LiCor, Cat # 92970020), then again purified. DTPA-antiGD2-IR800 was radiolabeled with In-111 using the Nalla et al.^40^ method for radiolabeling with minimal modifications.

### 2.3 Tissue Phantoms

Porcine liver, lung, fat, and muscle were purchased from Animal Biotech (Cat # LVWOGB, LUS, FA, BW). Slices ranging from 0.2 to 5.0 cm in thickness were cut using a mandolin slicer or by placing blocks of tissue in 3D-printed frames of the specified thickness, then slicing off additional tissue [Fig. 5(b)].

Gelatin phantoms were made with methods adapted from Ref. 35. One TBST tablet (Fisher, Cat # 5247501EA) was dissolved in 470 mL of deionized water and heated with continuous stirring. Fifty grams of porcine gelatin (Sigma-Aldrich, Cat # G1890) was slowly added while heating to 50°C. The solution was then cooled to 35°C, then bovine hemoglobin dissolved in ${\mathrm{ddH}}_{2}O$ (Sigma, Cat # H2625, final concentrations 4.2%, 8.3%, or 16.7%) and 25 mL of 20% intralipid (Sigma, Cat # I141, final concentration 1%) were added. When applicable, IRDye800CW was added to the gelatin solution and thoroughly mixed for uniformity prior to solidification. Gelatin of the specified thickness was solidified in 0.5 cm Petri dishes and was then peeled off and placed on top of tumors, or it was poured to the specified thickness and solidified directly on top of TBTs or TLIs in 3D-printed phantom molds [Fig. 5(b)].

### 2.4 3D Printing

3D-printed molds were designed with Tinkercad software (Autodesk, 2024), exported as.stl files, and sliced to.ufp files for printing with UltiMaker Cura software v 5.4.0 (Fig. 5). TLIs were designed as hemispheres (100  μl, unless otherwise specified). Normal resolution settings were typically used, with an infill density of 20%, infill triangle pattern, shell thickness of 0.8  mm×1.0  mm, and with adhesion enabled. Fast-resolution settings were occasionally used for larger molds. Molds were then printed on UltiMaker 3 or S5 3D printers with black PLA filament (Matter Hackers Build Series) and default temperature settings.

### 2.5 Tumor-Like Inclusions

Indium-111 chloride (BWXT Cat #10009095) or IRDye800CW (Li-COR Cat #92970020) was added to 20% gelatin in water prior to cooling. The specified volume of TLI solution (typically 100  μL) was pipetted into TLI wells (empty inverted hemispheres of the same volume) at the base of the phantom molds (Fig. 5).

### 2.6 Detection of Gamma Decay and Fluorescence Signal

Gamma signal was measured using a handheld Neoprobe^®^ instrument (Mammotome, Cincinnati, Ohio), and near-infrared images were obtained using a SPY-PHI handheld near-infrared camera (Stryker, Portage, Michigan). Porcine tissues or gelatin phantom increasing from 0.1 to 5.0 cm thick were sequentially added or solidified over each TBT or TLI, then the maximum gamma signal was measured and a near-infrared image was acquired (Fig. 7).

Fluorescence images from the SPY-PHI were captured using a NIX HDMI capture card (model USBC-CAP60, Plugable Technologies, Redmond, Washington) and OBS video capture software (Open Broadcaster Software, open source). The fluorescent signal was quantified using ImageJ software (NIH) by drawing an ROI over TBT or TLIon a uniformly enhanced image (to best visualize the full TBT/TLI for accurate ROIs), then measuring the fluorescence intensity per pixel squared on the unenhanced (original) image. The background signal was subtracted to determine the mean fluorescent intensity (MFI) of each TBT or TLI. Any signal lower than the background was considered no signal.

### 2.7 Simulation of Intraoperative Molecular Imaging

Porcine body wall tissue blocks were cut to ∼15 to 20 cm in width and length and included ∼4  cm of skin, subcutaneous fat, and muscle. Three divots were cut at random into the muscle side and resected TBTs about 1 cm in diameter from prior experiments were placed into the divots. The body wall blocks were then carefully flipped over, resulting in skin on the top side, and TBTs buried at the bottom of the 4-cm-thick tissue block.

For white-light surgery simulations, a photograph was taken prior to flipping the tissue block, then was oriented to match the skin-side-up specimen. A blinded investigator then attempted to resect the embedded TBTs, either by comparing them with the photograph (white-light only simulation) or with the use of a Neoprobe^®^ and SPY-PHI camera.

### 2.8 Preparation of Figures

Graphs were prepared with GraphPad Prism v.10 (Domatics), 3D-printed mold schematics were generated with Tinkercad software, experimental schematics were created using Biorender, and figures were otherwise prepared using PowerPoint (Microsoft).

### 2.9 Quantification and Statistical Analysis

Data was processed using GraphPad Prism v.10 (Domatics). Gamma signal strength and fluorescence intensities were compared by two-way ANOVA, with the Sidak post hoc test, when appropriate. Autofluorescence and background fluorescence between organs were each compared using one-way ANOVA, and if significant, the Sidak post hoc test was used to compare the uptake of each organ to the tumor uptake. Surgical defect volumes between IMI and non-IMI groups were compared using an unpaired t test.

## 3 Results

### 3.1 Signal from Tracer-Bearing Tumors Can Be Measured Through Porcine Tissue Phantoms to Determine Detection Limits

While tumor models can be used to study IMI tracers *in vivo*, modifications to assess specific properties are limited. For example, xenografted tumors are often superficial, which limits the ability to assess the impact of overlying tissue (which occurs with human tumors) on the detection of IMI tracers. To simulate coverage of the tumor by overlying tissue, a tracer was administered to neuroblastoma-bearing mice, the TBTs were resected, and then placed under slices of porcine tissue (liver, fat, muscle, and lung). Gamma decay was measured with the handheld Neoprobe^®^, and near-infrared fluorescence was assessed with the SPY-PHI camera at baseline and as each thicker section of porcine tissue was placed on resected tumors (Fig. 6).

**Fig. 1:**
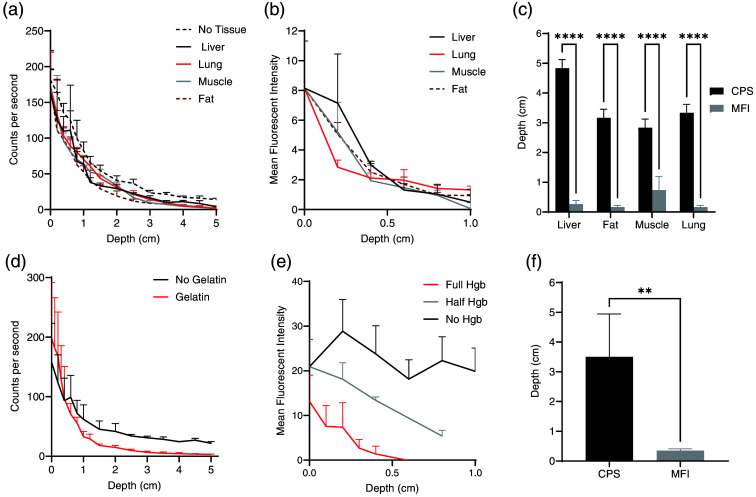
Signal from TBTs decreases as the thickness of the overlying phantom increases. (a) The gamma decay signal (in cps) of Indium-111 in TBTs decreases as thicker porcine tissue is placed between the TBT and Neoprobe^®^ (baseline signal is at a depth of 0 cm). (b) The fluorescence signal (in MFI) of IRDye800CW in TBTs decreases with porcine tissue between the TBTs and SPY-PHI camera (baseline fluorescence is at a depth of 0 cm, where there is no intervening tissue). (c) Comparison of the maximum tissue thickness (in mm) through which 5% of the baseline (0 mm) gamma decay from In-111 and fluorescence from IRDye800CW in TBTs can still be detected. (d) Gamma decay signal (in cps) from In-111 and (e) fluorescence (in MFI) from IRDye800CW in TBTs similarly decreases with thicker gelatin phantom between the TBTs and the Neoprobe^®^ or SPY-PHI. (f) Comparison of the maximum thickness of gelatin phantom through which 5% of the baseline (0 mm) gamma decay from In-111 and fluorescence from IRDye800CW can still be detected.

Gamma decay signal from TBTs decreased exponentially with increasing thicknesses of each of the porcine tissues [Fig. 1(a)]. This decay was more substantial with each type of tissue than with only intervening air (p<0.001 between air and each tissue, p=ns between tissue types). To avoid artificially inflating the maximum thickness for signal penetration at the asymptotic tail, the tissue thickness through which at least 5% of the baseline signal could be detected was compared [Fig. 1(c)]. The Neoprobe^®^ could detect 5% of the maximum gamma decay signal from the tracer in the tumor through at least 5 cm of air, 4.3±0.29  cm of the liver, 3.0±0.5  cm of fat, 3.2±0.29  cm of muscle, and 3.5 cm of the lung, with detection through significantly thicker liver than other tissues [p<0.005; Fig. 1(c)].

The attenuation of near-infrared fluorescence through tissue was not significantly different between tissue types [Fig. 1(b)]. Tumors could be visualized (5% of baseline fluorescence) through up to 0.27±0.12  cm of the liver, 0.13±0.12  cm of the fat, 0.33±0.12  cm of the muscle, and 0.07±0.12  cm of the lung, with no significant difference between the tissue types [Fig. 1(c)]. Gamma decay was detectable through significantly thicker tissue than fluorescence for all tissue types (p<0.0001), demonstrating the benefit of gamma decay detection in finding non-superficial tumor nodules.

### 3.2 Modifiable Gelatin Phantoms Can Elucidate the Effect of Overlying Tissue Properties on Signal Penetration

Although porcine tissues mimic organs, they are not homogenous, have post-mortem changes including decreased and deoxygenated blood, and do not include radioactive nor fluorescent background signals that could be generated by non-specific accumulation of tracer. Gelatin phantoms enable a more homogenous phantom that could incorporate a background signal. Similar to published methods, 10% gelatin phantoms in TBST were made with 0.170  μM hemoglobin and 1% intralipid to simulate background absorbance and light scattering properties of normal human tissue. Excised TBTs were placed in custom 3D-printed molds (Fig. 5), and then gamma decay and fluorescence were again measured as thicker slices of gelatin were placed on top of the tumors.

Similar to overlying tissue, increasing thicknesses of gelatin correlated with decreased gamma decay detection with the Neoprobe^®^, with a slightly greater decrease than with distance/air alone [Fig. 1(d)]. Gamma decay was still detectable, however, through up to 3.6±1.3  cm of gelatin phantom [Fig. 1(f)]. Also, in agreement with the porcine tissue model, fluorescence from TBTs attenuated with increased tissue thickness and 5% of the baseline fluorescence was visible through up to 0.35±0.06  cm of gelatin, significantly lower than the thickness through which gamma decay could be detected [p<0.005; Figs. 1(e) and 1(f)]. To demonstrate modification of the gelatin to mimic different tissue properties, fluorescence attenuation was also measured through gelatin with half the normal concentration of hemoglobin (0.085  μM) and without any hemoglobin, resulting in significantly different attenuation with increasing gelatin thickness [Fig. 1(e)].

**Fig. 2:**
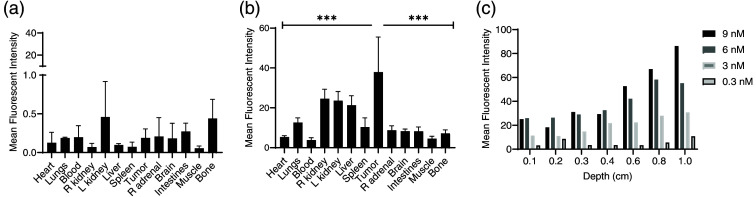
Autofluorescence and non-specific fluorescence occur in non-tumor tissues and can be mimicked in a gelatin phantom. (a) Near-infrared autofluorescence (when no tracer has been given) is minimal in all tissues, with MFI less than 1. (b) Non-specific fluorescence (from DTPA[In-111]-antiGD2-IR800 tracer outside of GD2-expressing tumors) occurs and could obscure tracer specifically accumulated in GD2-expressing tumors. (c) IRDye800CW can be added to gelatin phantoms to mimic the non-specific fluorescence observed in non-tumor tissue *in vivo*.

Using the neuroblastoma mouse model, background fluorescence from tumors and organs was measured without tracer administration (background) and after tracer distribution (non-specific accumulation). Without a tracer, there was negligible fluorescence from any of the organs, including the tumor, demonstrating very low auto-fluorescence in the near-infrared range [Fig. 2(a)]. This was comparable to the gelatin phantom, which similarly displayed a minimal amount of background fluorescence in the near-infrared range. Days after tracer administration when accumulation was highest in the tumor (as expected), non-tumor organs did demonstrate fluorescence and gamma decay from non-specific accumulation [Fig. 2(b)].

To mimic non-specific fluorescence in overlying tissue, gelatin with dilutions of IRDye800CW from 0.3 to 9 nM was made in a custom 3D-printed mold with thicknesses ranging from 1 to 10 mm [Figs. 2(c) and 5(b)]. Fluorescence for dilutions between 0 and 0.29 nm was similar to the non-specific fluorescence observed in the muscle (low fluorescence) while dilutions between 1.8 and 6.4 nm overlapped with the non-specific fluorescence in the liver (high fluorescence) after tracer administration.

### 3.3 Tumor-Like-Inclusions Can Be Adjusted to Study the Effects of Tumor Variation on Signal Detection

As xenograft tumors vary unpredictably in size and viability, it is difficult to assess the effect of size and epitope (i.e., GD2) expression on fluorescence and gamma decay signal. To study the effect of variable radioactivity and fluorescence from TBTs (to mimic the combination of epitope expression and tracer accumulation), as well as tumor size, TLIs were generated. Mold forms were 3D-printed with specifically sized empty hemispheres for TLIs on the upper surface of the base plate (Fig. 5). The base plate was enclosed within steps of 1 to 5 mm to pour gelatin of precise thicknesses. TLIs were then generated by diluting In-111 or IRDye800CW in a warm gelatin solution (with similar radioactivity or fluorescence to the tumors), and then solidified in the 3D-printed molds.

**Fig. 3:**
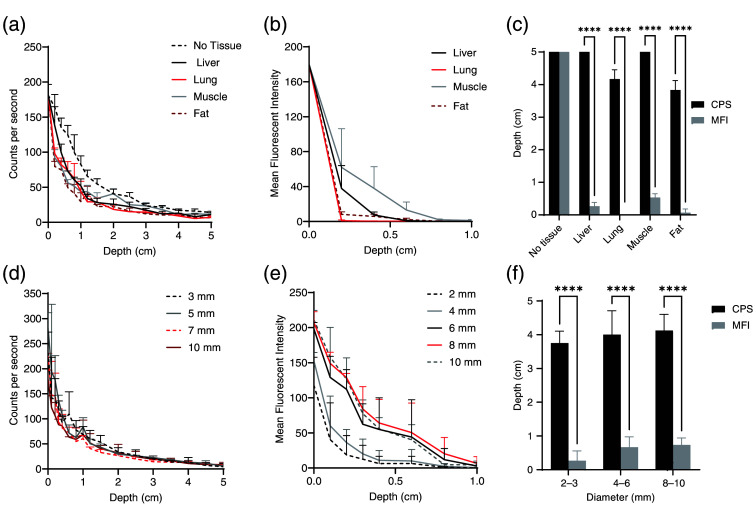
Signal from TLIs also decreases as tissue phantom increases in thickness between TLIs and detectors. (a) Gamma decay signal from In-111 in TLIs decreases as additional porcine tissue is placed between the TLI and Neoprobe^®^. (b) Fluorescence intensity from IRDye800CW in TLIs also decreases with more intervening porcine tissue. (c) At least 5% of the baseline gamma decay signal (signal measured with no intervening tissue nor gelatin) can be detected through significantly thicker tissue than at least 5% of the uncovered fluorescence signal. (d) Gamma decay from In-111 in TLIs decreases with intervening gelatin phantom in a similar pattern, regardless of the size of the TLI. (e) Fluorescence signal decay through gelatin is not visible through thicker gelatin phantom when TLIs are larger (p=ns). (f) At least 5% of the baseline gamma signal penetrates through more gelatin phantom than the fluorescence signal, regardless of the TLI size.

To confirm similar detection properties to TBTs, radioactivity and fluorescence were measured from TLIs through increasing thicknesses of the phantom. Signal attenuation through porcine tissue was similar, for gamma decay and NIR fluorescence [Figs. 3(a) and 3(b)]. At least 5% of the maximum gamma decay signal was detected through at least 5 cm of the liver, 3.8±0.3  cm of the fat, 5 cm of the muscle, and 4.2±0.3  cm of the lung, and the signal penetrated through more liver and muscle than the lung and fat (p<0.0001). TLI diameter did not affect the rate of gamma signal decay [Fig.  3(e)]. This was also significantly thicker tissue than that through which at least 5% of the NIR fluorescence could be detected for each tissue type (p<0.0001), including 0.27±0.12  cm of the liver, 0.07±0.12  cm of the fat, 0.53±0.12  cm of the muscle, and <0.2  cm of the lung [Figs. 3(b) and 3(c)].

TLI size was then modified to demonstrate the effect of tumor size on detection. Gamma decay signal from TLIs again decreased with increasing thicknesses of gelatin phantom, but up to 5% of the maximum signal remained detectable through 4.0±0.5  cm, with no difference due to TLI size [Figs. 3(d) and 3(f)]. TLIs were also generated with increasing diameter and brightness. A significant difference in fluorescence attenuation through gelatin phantom was detected for different size TLIs (p<0.0001). At least 5% of baseline fluorescence was seen through 0.27±0.29  cm with 2-mm diameter TLIs, 0.67±0.31  cm with 4-mm diameter TLIs, and through 0.73±0.21  cm with 8- to 10-mm diameter TLIs [Figs. 3(e) and 3(f)].

### 3.4 Simulation of Intraoperative Molecular Imaging for Identification of Tumors Hidden in Porcine Tissue

**Fig. 4:**
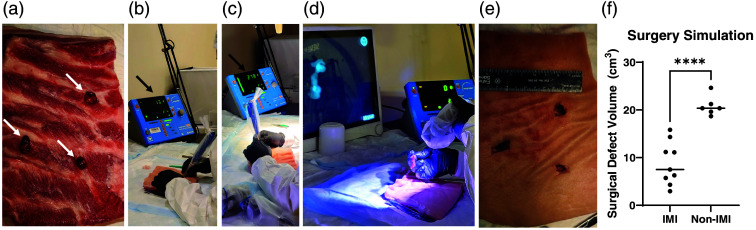
(a) Tracer-bearing xenograft tumors (arrows) were embedded on the back side of a section of the porcine body wall, which was then carefully turned over so the skin was on top. (b) For IMI experiments, a blinded investigator used the Neoprobe^®^ to determine the location of the embedded tumors (arrow pointing to counts of 13 cps). (c) As the investigator dissected closer to the tumor, the gamma decay signal increased (arrow pointing to increased counts of 273 cps). (d) Once nearly exposed, the investigator used the SPY-PHI camera to assess the fluorescence of the tumor (fluorescent image in background), which is then shown to have clear margins when removed. (e) After tumors were resected, surgical defects in the porcine tissue were measured and (f) defects were smaller when IMI was used ($8.82\pm 4.51\;{\mathrm{cm}}^{3}$) versus when IMI was not used ($20.82\pm 2.1\;{\mathrm{cm}}^{3}$; p<0.0001).

To further investigate the utility and practice of IMI, a blinded investigator attempted to resect TBTs that had been embedded on the back side of a porcine abdominal wall section (three tumors per trial). Using the Neoprobe^®^ and then the SPY-PHI allowed the investigator to clearly visualize and “resect” the TBTs (Fig. 4). Non-IMI guided resection (where the blinded investigator was shown an image of the tumors within the back side of the tissue) was also attempted. The tissue defect created during resection with IMI was significantly smaller than that created with unguided, white-light-only resection ($8.82\pm 4.51\;{\mathrm{cm}}^{3}$ versus $20.82\pm 2.1\;{\mathrm{cm}}^{3}$; p<0.0001; Figs. 4(f) and 8).

## 4 Discussion

Despite the clinical importance of safe and complete resections of solid tumors, surgeons still primarily rely on visualization and palpation to identify tumors and distinguish them from normal or fibrotic tissue, as they have for decades. Tumors can be challenging to find during surgery due to shifts in the position of soft tissues or organs (i.e., lung and intestine) when compared with the preoperative images, overlying tissue, or lack of detection by imaging. IMI, however, can enable the surgeon to find tumor tissue during surgery and distinguish it from non-cancerous tissue, potentially enabling a safer and more complete resection.^23^^,^^41^ IMI tracers target tumor tissue with radioisotopes (RGS) and/or fluorophores (FGS) that enable detection and visualization of tumor margins, using already commonly used tools.^16^^,^^23^^,^^42^^–^^44^ Radioisotopes enable excellent depth of detection but limited spatial resolution, while fluorophores can only be visualized through a few millimeters of tissue but provide excellent visualization of the target tissue.

While research into IMI tracers has expanded, an understanding of the limits of detection is lacking. Phantom studies enable some assessment of the impact of tumor and tissue properties, through modification of TLI size and tracer concentration, inclusion of scattering and absorbing molecules in solidified polymer solutions, and modification of molds for phantom shapes.^32^^,^^33^^,^^35^ These models, however, are limited in the ability to easily customize the phantom mold shape, precisely size and place TLIs, and by a lack of direct comparison with actual tissues. Here, we investigate those limits for both gamma decay from indium-111 and fluorescence from IRDye800CW, as well as describe methods for measuring additional detection properties.

To closely replicate the real-world *in vivo* use of an IMI tracer, a dual-labeled IMI tracer targeted to the overexpressed GD2 epitope (DTPA[In-111]-antiGD2-IR800) was used in a xenograft model of neuroblastoma. Neuroblastoma tumors were grown for 5 weeks in nude mice to achieve a diameter of around 1 cm.^39^ Tracer was administered, and after 4 days for accumulation and serum clearance, TBTs were resected and used for further experiments.^23^^,^^45^ Gamma decay and near-infrared (NIR) fluorescence of tracer in tumors was measured with commonly used intraoperative tools, the Neoprobe^®^ and SPY-PHI, at baseline and with intervening porcine tissue to simulate the basic physical properties of the human tissue.

As expected for radioactivity, the gamma decay signal decreased exponentially with increasing distance from the TBTs [Fig. 1(a)]. The attenuation of gamma decay through tissue is known to depend on multiple factors, including the particle energy, as well as the chemical composition and density of the tissue.^46^ Concordantly, the gamma decay signal from the TBTs was slightly higher when there was no intervening tissue versus each type of porcine tissue, though no difference was detected between the tissue types. Because of the asymptotic tail of the signal curve, the maximum depth of detection was defined as the depth at which at least 5% of the maximum (uncovered) signal was observed. Gamma decay from tumors was detectable through at least 5 cm of the air, 4.3±0.29  cm of the liver, 3.0±0.5  cm of the fat, 3.2±0.29  cm of the muscle, and 3.5 cm of the lung, with a detection through significantly thicker liver than other tissues [p<0.005; Figs. 1(a) and 1(c)]. Porcine tissue, however, is heterogeneous, and obtaining precise slices less than 5-mm thick is technically challenging, contributing to relatively large standard deviations.

NIR fluorescence brightness similarly declined with increasing intervening tissue [Figs. 1(b) and 7]. As the SPY-PHI held a consistent distance from the tumor, regardless of intervening tissue, the fluorescence signal decrease was not measured for the increased distance of intervening air. No difference was observed in the fluorescence decay curves with different tissue types. Up to 5% of maximum fluorescence from TBTs was visualized by the SPY-PHI through up to 0.27±0.12  cm of the liver, 0.13±0.12  cm of the fat, 0.33±0.12  cm of the muscle, and 0.07±0.12  cm of the lung, with no significant difference between the tissue types [Figs. 1(b) and 1(c)]. Though no significant difference was seen, some variability in signal penetration could be caused by the effect of tissue on light absorbance and scatter, as well as tissue heterogeneity. This short penetrance demonstrated the benefit of dual-labeled tracers, as gamma decay enables detection through an additional 3 to 4 cm of tissue [Fig. 1(c)].

Although quite similar to normal human tissue, porcine tissue has multiple limitations as a tissue phantom: Thicknesses are imprecise, the tissue is not homogenous, there are post-mortem changes (de-oxygenation and loss of blood), and the tissues lack nonspecific gamma decay and fluorescence signal from the administered tracer.^35^^,^^47^ Gelatin phantoms, however, enable the generation of precise thicknesses, homogeneity, and the inclusion of gamma decay particles and fluorophores to simulate autofluorescence and non-specific tracer accumulation seen in various tissues.^35^ A modified gelatin phantom recipe was used, which included 10% gelatin in TBST, 0.170  μM hemoglobin to mimic tissue absorbance, and 1% intralipid to mimic tissue scatter. Phantoms were used shortly after generation, so ${\mathrm{NaN}}_{3}$ was not included to prevent bacterial contamination. While bilirubin and PPIX are sometimes included in gelatin phantoms to simulate autofluorescence with peaks of 520/570  nm and 640 to 700 nm, respectively, they were not included here, as IRDye800CW fluoresces at much longer wavelengths (792 nm peak).^48^^,^^49^ Indium-111, IRDye800CW, or other isotypes and fluorophores could easily be added as well, to simulate various tissue characteristics and their impact on the use of IMI.

**Fig. 5:**
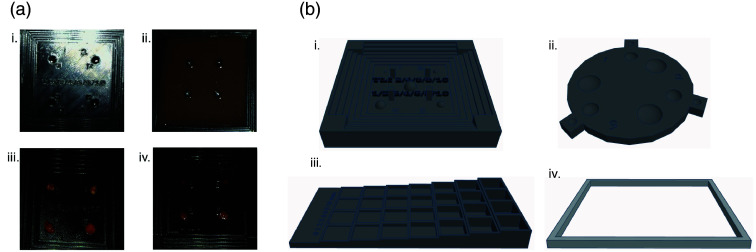
(a) Custom 3D-printed molds with (i) tumor-like inclusions exposed and (ii) under gelatin tissue phantom as well as (iii) TBTs exposed and (iv) under gelatin tissue phantom. (b) TinkerCAD schematic of custom 3D-printed molds including (i) stadium style plate for tumors and TLIs under gelatin, (ii) round style plate for tumors and TLIs under gelatin, (iii) step-style plate for measuring background signal from gelatin with dilutions of IR800, and (iv) a hollow frame (2, 4, 6, 8, or 10 mm tall) for cutting porcine tissues to specific thicknesses.

Using gelatin phantoms instead of porcine tissue, gamma decay signal from tumors exponentially decreased with distance in a similar manner, supporting its use in future phantom work [Fig. 1(d)]. Also similarly, the decrease in signal was slightly greater with intervening gelatin than with no intervening tissue [Fig. 1(d)]. While porcine tissue needed to be carefully cut to the appropriate thickness and lacks homogeneity, precise thicknesses of homogenous gelatin could be poured into 3D-printed molds with pre-determined steps of increasing thickness (Fig. 5). At least 5% of maximum gamma decay was detected through 3.6±1.3  cm of gelatin while at least 5% of the NIR fluorescence of the tumors could only be detected through 0.35±0.06  cm of gelatin [Figs. 1(d)–1(f)]. The impact of hemoglobin absorption of NIR light of fluorescence signal from the tracer was also suggested by significantly different fluorescence attenuation through gelatin without, with half the concentration of, and with the full amount of hemoglobin, similar to prior phantom work with varying hemoglobin concentrations [Fig. 1(e)].^33^ A shorter depth of detection relative to other phantom studies is likely due to the use of the SPY-PHI camera, which is widely used intraoperatively but optimized for the fluorescence properties of ICG, not IRDye800CW.^33^ No decrease in fluorescence was seen with increasing thicknesses of gelatin without hemoglobin, as light was minimally absorbed by gelatin alone.

To assess tissue autofluorescence detected by the SPY-PHI camera in the near-infrared wavelength range, SPY-PHI images were taken of organs removed from mice that had not received any tracer. As expected for near-infrared wavelengths, negligible autofluorescence was seen in all measured organs [Fig. 2(a)]. For actual use of IMI tracers, however, some tracers will non-specifically accumulate in non-tumor tissue, leading to a higher background signal than autofluorescence alone. To measure the non-specific background fluorescence from the tracer with IRDye800CW with the SPY-PHI camera, fluorescence images were also taken of organs resected 4 days after tracer administration (the timing used for IMI), and average fluorescence intensity per pixel^2^ was measured for each organ, with the greatest non-specific fluorescence seen in the liver and kidneys [Fig. 2(b)]. Fluorescence from dilutions of IRDye800CW in gelatin was also imaged and quantified to correlate with tissue background signal for use in future phantom experiments [Figs. 2(c) and 5(biii)]. Though the background fluorescence seen in each organ would be dependent on the fluorophore and camera, this method could be applied to other tracers and fluorescence cameras as well. Bilirubin, PPIX, or other fluorophores could also be used to simulate autofluorescence and background fluorescence for tracers that fluoresce at shorter wavelengths.

As with porcine tissue, TBTs cannot be easily modified to assess the impact of target expression/tracer accumulation and tumor size. Tracer density and dimensions, however, can be set and modified with TLIs, pieces of gelatin that include IRDye800CW, In-111, or other signal moieties. Specifically, TLI dimensions can be precisely determined using 3D-printed molds, while tracer density can be adjusted by mixing in different concentrations of In-111, IRDye800CW, or other tracers.

Signals from TLIs demonstrated similar decay through porcine tissue and gelatin phantom to signal from TBTs [Figs. 3(a)–3(c)]. To demonstrate the modification of characteristics, TLIs 2 to 10 mm in diameter were compared as well [Figs. 3(d)–3(f)]. With a similar baseline total gamma decay to TBTs, the TLI diameter did not affect the decrease in signal with increasing gelatin thickness, with 5% of the maximum signal detectable through 4.0±0.5  cm. When the size and brightness of TLIs were increased, a significant difference in fluorescence attenuation through gelatin phantom was observed (p<0.0001), with smaller and dimmer TLIs seen through 0.27±0.29  cm, while larger and brighter TLIs were seen through 0.73±0.21  cm. Future work could more thoroughly investigate the impact of TLI parameters, such as depth, spread, In-111 density, and IRDye800CW density.

RGS can first be used to detect the location of the tumor through 3 to 5 cm of tissue, then direct dissection towards the tumor. Once close to the tumor (within mm), borders can be visualized with FGS, to precisely and safely resect the tumor. With dual-labeled tracers, the techniques of RGS and FGS can be used in an iterative and complementary process, as the surgeon alternates between them for dual confirmation at different depths. For a comprehensive analysis, it is important to understand the full parameters of gamma detection (including in the mm range), though we anticipate this would not be the primary technique used at superficial depths.

To simulate IMI use in surgery, TBTs were buried beneath a large piece of porcine body wall [Fig. 4(a)]. A blinded investigator was tasked with using the Neoprobe^®^ and SPY-PHI to find and remove these tumors while making the smallest incision and limiting the dissection as much as possible [Figs. 4(b)–4(d) and 8]. This is important, as it stimulates the attempt to preserve surrounding normal tissue. As the investigator neared each TBT, gamma decay detected by the Neoprobe^®^ increased [Figs. 4(b) and 4(c)], and fluorescence became visible as the investigator came within millimeters of the tissue, defining the margins. The investigator was able to find and remove the tumors, creating significantly smaller tissue defects than when attempting an unguided version of the “surgery” ($8.82\pm 4.51\;{\mathrm{cm}}^{3}$ versus $20.82\pm 2.1\;{\mathrm{cm}}^{3}$; p<0.0001; Figs. 4(f) and 8). This method could also be used to teach surgeons how to use and practice IMI. The use of porcine tissue is advantageous relative to prior work in gelatin or silicone molds, as the tissue feels more similar to what surgeons encounter intraoperatively.^31^^,^^33^ This work also expands on phantom work with dual-labeled tracers.^31^ This trial demonstrated that our dual-labeled tracer allowed the investigator not only to find the lesions with greater ease, but to remove them without removing or damaging as great of an amount of healthy tissue.

A major goal for using intraoperative IMI is to overcome the challenges in locating tumors and identifying tumor margins during surgery, where shifting of tumor position, deformation of tumor and its surrounding benign tissues, and tumor invasion on neighboring tissues occur. A limitation of our current model is the stationary nature of the TBT relative to the normal tissue, so preoperative “imaging” more closely locates tumor tissue than actual preoperative imaging. Additionally, as TBTs are placed within tissue block defects and do not grow into them, “tumor margins” are very easily defined with white light alone. Future models should incorporate the shifting and deformation of tissues, as well as the tumor invasion seen intraoperatively. Various tumor shapes, such as those with spiculated edges, could also be easily generated in TLIs with these methods. The porcine tissue block model with embedded TBTs, however, is an excellent initial training tool for the use of IMI, and future work could also assess the time to resect the tumor.

In summary, gamma decay from an indium-111-based IMI tracer can be detected through 2.8 to 4.8 cm of tissue with the Neoprobe^®^ (depending on the tissue type), while near-infrared fluorescence from an IMI tracer with IRDye800CW can be visualized through only 2 to 8.7 mm of tissue using the SPY-PHI camera, though it is easy to visualize at superficial depths. Gelatin phantoms and TLIs created in custom 3D-printed molds enable precise modification of tumor and overlying tissue characteristics, including radioactive or fluorescent background signal, tissue thickness, TLI dimensions, and TLI signal strength. Here, we demonstrated its use for assessing a particular tracer, DTPA[In-111]-antiGD2-IR800 for neuroblastoma, but those methods can be applied to any radioactive or fluorescent tracer. Similarly, while the Neoprobe^®^ and SPY-PHI are commonly used intraoperatively, these methods could also be applied to other detection systems. This would be particularly important for fluorescence cameras, as detection properties depend on the overlap of excitation and emission wavelengths between tracers and cameras. These data also further reinforce the benefits of dual-labeled tracers, as gamma decay enables depth of detection, while NIR fluorescence enables excellent visualization and resolution when seeing the tumor directly.

## 5 Appendix: Supplementary Figures

**Fig. 6:**
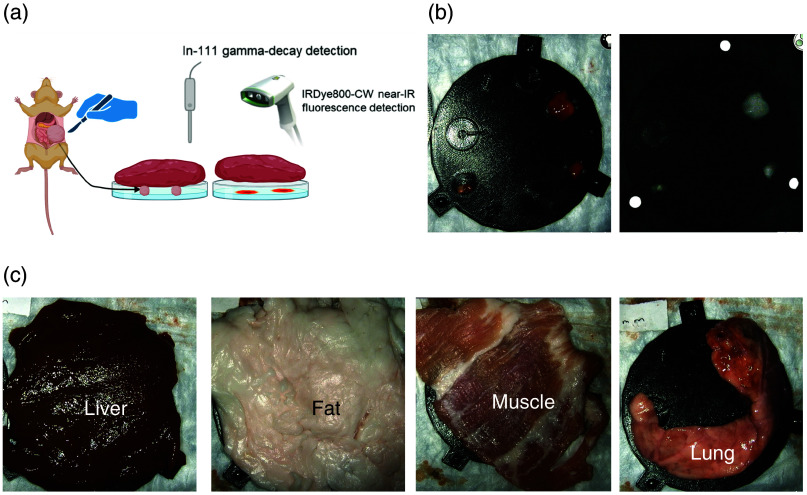
(a) Schematic of TBT resection, placement on plates, coverage by porcine tissue, and measurement by Neoprobe^®^ and SPY-PHI. (b) Uncovered white-light (left) and NIR fluorescent (right) images of TBTs on 3D-printed plates. (c) Sample TBT coverage by each type of porcine tissue.

**Fig. 7:**
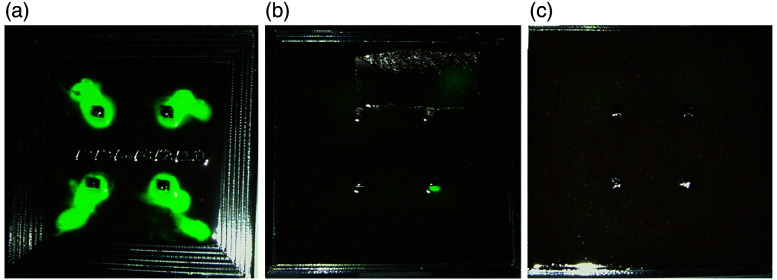
Overlay images of fluorescence of IR800 containing tumor-like inclusions, at depths of (a) 1 mm, (b) 6 mm, and (c) 10 mm of gelatin tissue phantom.

**Fig. 8:**
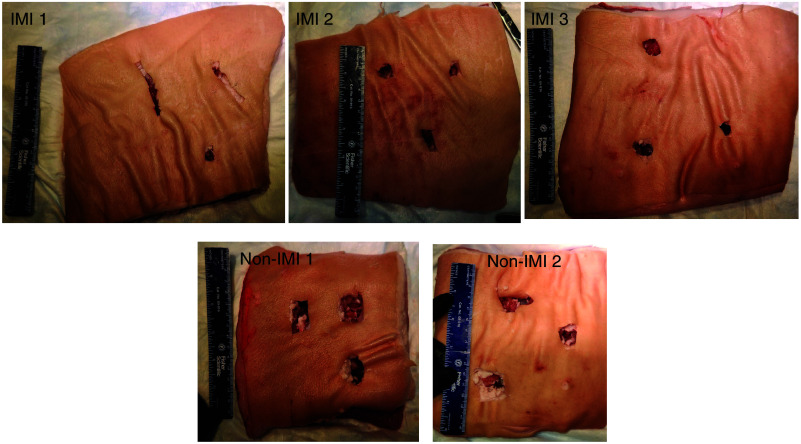
Images of surgical defects for simulated tumor nodule resection with IMI (top) and without IMI (bottom).

These further describe the techniques used to measure detection of In-111 and IRDye800CW through tissue and gelatin phantoms (Figs. 5–8).

## Data Availability

All data reported, any additional information required to reanalyze the data shown in this paper, and 3D printing code files are available from the lead contact upon request. Request for additional information and resources should be sent to Gary Kohanbash, PhD (gary.kohanbash2@chp.edu).
